# Immune recall enhances cross-reactive antibody longevity after a large wave of SARS-CoV-2 breakthrough infection

**DOI:** 10.1038/s41392-024-01926-w

**Published:** 2024-08-21

**Authors:** Dan Li, Qingfei Chu, Kang Li, Lanjuan Li, Yiming Shao

**Affiliations:** 1 State Key Laboratory for Diagnosis and Treatment of Infectious Diseases, National Clinical Research Center for Infectious Diseases, National Medical Center for Infectious Diseases, Collaborative Innovation Center for Diagnosis and Treatment of Infectious Diseases, The First Affiliated Hospital, Zhejiang University School of Medicine, Hangzhou, 310003 China; 2 National Key Laboratory of Intelligent Tracking and Forecasting for Infectious Diseases, National Center for AIDS/STD Control and Prevention, Chinese Center for Disease Control and Prevention, Beijing, 102206 China

**Keywords:** Infectious diseases, Infectious diseases

**Dear Editor**,

Although coronavirus disease 2019 (COVID-19) is no longer deemed a “Public Health Emergency of International Concern”, the global transmission dynamics of SARS-CoV-2 have shifted due to vaccination, emergency of virus variants, and pre-existing coronavirus immunity.^1–3^ It remains unclear how cross-reactive and pre-existing humoral immunity might impact the effectiveness of vaccines and antibodies against new viral mutations.^4,5^ This study seeks to elucidate the cross-reactivity of coronavirus antibodies induced by previously unencountered Omicron BA.5/BF.7 variants in the breakthrough infection (BTI) population.

We enrolled 59 participants, with an average age of 34 (range: 22–67) years, comprising 42% males. Notably, 97% of people have received the inactivated COVID-19 vaccine (BBIBP-CorV or CoronaVac), of which 54% have received booster shots (third or even fourth doses). The cohort consisted of 50 patients with convalescent COVID-19 (40 with mild symptoms, 10 with moderate symptoms) and 9 SARS-CoV-2 naïve controls (who were all vaccinated but had not been infected when they were first recruited) (Fig. 1a). Moderate individuals exhibited more respiratory symptoms, while those with mild cases experienced predominantly gastrointestinal issues. In our cohort, most infections (from December 2022 to January 2023) were presumed to be the Omicron-BA.5/BF.7 strain, based on prevailing variants in China.

**Fig. 1:**
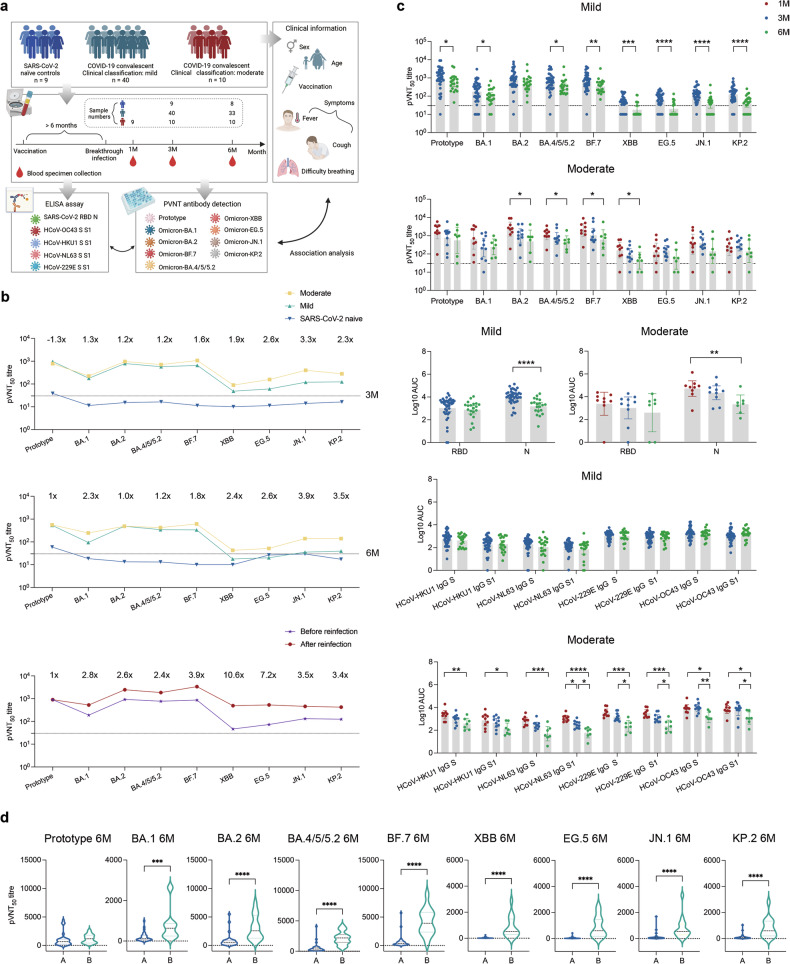
**a** Flowchart of this study. It shows in detail the number of samples collected at each time point. A total of 122 cases underwent subsequent ELISA and PVNT testing (Figure created using BioRender.com). **b** Trends of pVNT_50_ titres against SARS-CoV-2 Prototype and eight Omicron variants based on sera collected from study cohort. Geometric mean titre (GMT) of pVNT_50_ is shown as dots joining connecting lines between variants. Samples are colored by different groups. The value shown above is the ratio of moderate group to mild group or the ratio of after reinfection group to before reinfection group. **c** The top two pictures represent the comparison of nAbs in mild and moderate infection groups at 1, 3 and 6 months after breakthrough infection (BTI). The dotted line represents the lowest limit (pVNT_50_ = 30) of the PVNT assay. Each point in the graph represents a sample and the bar value is represented by geometric mean with 95% CI. The middle two pictures represent the comparison of binding antibody levels in mild and moderate infection groups at 1, 3 and 6 months after BTI. The bottom two pictures represent the HCoV-OC43, HCoV-HKU1, HCoV-NL63 and HCoV-229E IgG S- and S1-binding antibody at three follow-up time points (1, 3, 6 months). **d** Comparison of nAbs in people with reinfection and those without reinfection at 6-month follow-up. A stand for without reinfection, B stand for reinfection. Statistical comparisons were made using two-tailed, nonparametric Mann–Whitney U-tests and two-tailed, nonparametric Uncorrected Dunn’s Kruskal–Wallis tests for numerical data. *p < 0.05; **p < 0.01; ***p < 0.001; ****p < 0.0001. Other unmarked ones are ns, not significant. 1 M, 1 month after COVID-19 recovered; 3 M, 3 months after COVID-19 recovered; 6 M, 6 months after COVID-19 recovered. AUC, area under the curve

We investigated antibody responses in individuals recovering from mild and moderate COVID-19, as well as a SARS-CoV-2 naïve control group, over 1, 3, and 6 months postrecovery. Using pseudovirus neutralization test (PVNT), we assessed neutralizing antibodies (nAbs) against nine SARS-CoV-2 strains: Prototype (PT), and Omicron variants (BA.1, BA.2, BA.4/5/5.2, BF.7, XBB, EG.5, JN.1 and KP.2). Post-recovery patients, irrespective of COVID-19 severity, maintained elevated levels of nAbs compared with SARS-CoV-2 naïve controls at both 3 and 6 months. Overall, the antibody level of moderate cases was higher than that of mild cases, but there was no statistical difference except for the nAbs level of JN.1 at three months of recovery. We may attribute it to the small sample size of this study. The geometric mean titre (GMT) line chart also showed a slightly higher nAbs level in moderate cases (Fig. 1b). Intriguingly, for XBB, EG.5, JN.1 and KP.2 variants, emerging after BF.7, participants displayed relatively lower nAb levels. It was worth mentioning that through the 6-months follow-up study on the unexposed variants, though JN.1 and KP.2 are recently emerged strains, we observed relatively higher levels of nAbs of these two variants than that of the previous XBB and EG.5 at the three-month recovery after COVID-19. But at the six-month follow-up time points, in the re-infected donors, despite nAbs to all tested strains were increased, the response to XBB and EG.5 were stronger than JN.1 and KP.2 which may due to the XBB epidemics in China between the two follow-up time points. The most probably reason for this phenomenon may be that virus strains derived from the same evolutionary branch had similar serological trend. Based on this, we could speculate that the humoral immune response trend to the future virus stain could be predicted according to the previous exposed virus on the same evolutionary branch (Fig. 1b).

Over time, nAbs exhibited a more pronounced decline in mild cases compared to moderate ones, possibly indicating heightened immune activation in moderate subjects, or it may be limited by the small sample size of our moderate subjects (Fig. 1c). Our findings were supported by enzyme-linked immunosorbent assay (ELISA) tests for S-RBD and N IgG levels (Fig. 1c). Correlation analysis corroborated a positive association between RBD/N binding abilities and PVNT results (data not shown).

After COVID-19 recovered, the levels of nAbs and binding antibodies decreased. Interestingly, seasonal coronavirus did not decrease significantly in the mild population but decreased in different degrees in moderate subjects (Fig. 1c). In addition, we evaluated the pre-existing seasonal coronaviruses antibodies and explored the correlation between seasonal coronaviruses and SARS-CoV-2. It is shown that individuals from moderate cases exhibited significantly higher levels of HCoV-OC43 S and S1 antibodies compared to those with mild cases. In addition, HCoV-OC43 S and S1 antibodies are positively correlated with SARS-CoV-2 S-RBD, Prototype, EG.5 and JN.1 (data not shown). Collectively, our results suggested that pre-existing seasonal coronaviruses may influence SARS-CoV-2 antibody responses, possibly revealing synergistic antibody reactivity between HCoV-OC43 and SARS-CoV-2.

In addition, at 6 months post-recovery of BTI, individuals maintained a certain antibody level, yet the decline in antibodies heightened the risk of reinfection with different variants. Among study cohort, 15 experienced reinfection (3 people were from the moderate group and 12 people were from the mild group). Those with reinfection exhibited notably higher nAbs against Omicron variants, while their ability to neutralize the original strain mirrored that of individuals without reinfection (Fig. 1d).

Our study population underwent a prolonged follow-up period, particularly considering China’s enduring strict lockdown measures and widespread vaccination efforts. The infection dynamics markedly differ from those observed internationally. Many individuals in our cohort were never infected before having received SARS-CoV-2 vaccinations. Despite these preventive measures, a sudden surge in BTI occurred within a short timeframe. The simultaneous detection and analysis of multiple strains represent notable strengths of this study. While the XBB variant of the coronavirus triggered a new epidemic wave globally, in China, many cases were still attributed to the BA.5/BF.7 strains. Our longitudinal study highlighted a predominance of XBB in secondary reinfection, that further indicated a major shift in infection subtypes over time. In addition, we also explored the newly emerging strains of EG.5, JN.1 and KP.2. Compared with the subjects without secondary reinfection, the population after reinfection also had higher nAbs to these three strains.

Nevertheless, our study has limitations. The study suffered from insufficient sample sizes of moderate cases and SARS-CoV-2 naïve controls. We did have serum samples from 1, 3, and 6-month timepoints after COVID-19 recovery but we didn’t collect nasopharyngeal swabs from subjects during their infection period for sequence testing. In addition, due to lack of background information of pre-existing seasonal coronavirus before BTI, we could only analyze the changing trend of seasonal coronavirus after BTI during the follow-up period.

In conclusion, this study revealed the dynamics and influencing factors of cross-reactive nAbs responses to multiple SARS-CoV-2 variants in a cohort with vaccination and BTI through long-term follow-up. Stronger nAbs are preferred in patients with moderate COVID-19 diseases and HCoV-OC43 infections compared with those without. Broader nAb tend to cover the unexposed variants in the same or closer evolutionary branches with the BTI SARS-CoV-2 strains. The study sheds light on the preparation for a better strategy to deal with challenges future SARS-CoV-2 variants.

### Supplementary information

Supplementary Materials

## Data Availability

The de-identified dataset supporting the conclusions of this Article can be made available from the corresponding author upon reasonable request.
