# Supplementary material for: Immune recall enhances cross-reactive antibody longevity after a large wave of SARS-CoV-2 breakthrough infection
Source: Signal Transduct Target Ther. 2024 Aug 21;9:208. doi: 10.1038/s41392-024-01926-w (PMC11335868; doi:10.1038/s41392-024-01926-w)
Supplement: Supplementary file 1 — Supplementary Materials [file 41392_2024_1926_MOESM1_ESM.docx]

**Supplementary Materials for**

**Immune recall enhances cross-reactive antibody longevity after a large wave of SARS-CoV-2 breakthrough infection**

Dan Li^1,2#^, Qingfei Chu^1,2#^, Kang Li^2#^, Lanjuan Li^1*^, Yiming Shao^1,2*^

^1^State Key Laboratory for Diagnosis and Treatment of Infectious Diseases, National Clinical Research Center for Infectious Diseases, National Medical Center for Infectious Diseases, Collaborative Innovation Center for Diagnosis and Treatment of Infectious Diseases, The First Affiliated Hospital, Zhejiang University School of Medicine, Hangzhou, 310003, China.

^2^National Key Laboratory of Intelligent Tracking and Forecasting for Infectious Diseases, National Center for AIDS/STD Control and Prevention, Chinese Center for Disease Control and Prevention, Beijing, 102206, China.

^#^These authors contributed equally.

**^*^Correspondence to:**

Yiming Shao ([yishao16@zju.edu.cn](mailto:yishao16@zju.edu.cn)) and Lanjuan Li (ljli@zju.edu.cn)

**Materials and methods**

**Study population and design**

This study recruited 59 participants, including 50 patients with convalescent COVID-19 and 9 SARS-CoV-2 naïve controls. These SARS-CoV-2 naïve controls were all vaccinated but had not been infected when they were first recruited. Among the patients with convalescent COVID-19, 10 experienced moderate symptoms, while 40 had mild COVID-19, classified according to the 10th Edition of China's National Health Commission's guidelines. The difference between mild and moderate cases was primarily based on characteristic manifestations of COVID-19 pneumonia observed on imaging in patients with moderate COVID-19. The exclusion criteria for this study population included patients who had long-term use of immunosuppressants and those with clear concurrent lung infections caused by bacteria or other pathogens.

Serum samples were collected from January to July 2023 at three intervals: 1, 3, and 6 months post-recovery. The blood samples were also collected from SARS-CoV-2 naïve controls at these three time points. Comprehensive epidemiological data, including demographics, behavior, vaccination details, COVID-19 symptoms, and lab results, were gathered.

**Pseudotype virus and cells**

The pseudovirus preparation involved the co-transfection of 293 T cells with pcDNA3.1-S-COVID-19 (PT and Omicron variants BA.1, BA.2, BA.4/5/5.2, BF.7, XBB, EG.5, JN.1 and KP.2) and pVSV△G-Luc. Both plasmids carry the optimized spike (S) gene and the vesicular stomatitis virus backbone, respectively.

Huh-7 cells were cultured in complete Dulbecco's Modified Eagle Medium (cDMEM) supplemented with 1% penicillin-streptomycin and 10% fetal bovine serum (FBS). The cells were maintained at 37°C in a 5% CO2 environment.

**Pseudovirus neutralization test (PVNT)**

The experimental steps were as follows: Plasma was inactivated at 56°C for 30 min and centrifuged at 14,000 RPM for 1 min. Concurrently, 140 μL of complete Dulbecco's Modified Eagle Medium was added to each well of a 96-well plate. Duplicate samples of 11 μL human plasma were added to row H, columns 3-12, followed by triple dilution with an initial dilution of 1:20. Next, 50 μL SARS-CoV-2 pseudoviruses with a concentration of 1,300 TCID 50/mL were added to the plates, which were then incubated at 37°C for 45–90 min. Then, 100 μL Huh7 cells (20,000 cells/well) were added to the plates, followed by incubation at 37°C in a humidified atmosphere with 5% CO2 for 24 h. Chemical fluorescence readings were obtained using a luminometer, and the results were reported as half maximal inhibitory concentration of pVNT (pVNT_50_ titre).

**Enzyme-linked immunosorbent assay (ELISA)**

Recombinant proteins of SARS-CoV-2 RBD, SARS-CoV-2 N, HCoV-HKU1 S, HCoV-HKU1 S1, HCoV-NL63 S, HCoV-NL63 S1, HCoV-229E S, HCoV-229E S1, HCoV-OC43 S, or HCoV-OC43 S1 were obtained from Sino Biological. Then, 96-well microtiter plates (Thermo Fisher) were coated with 100 μL recombinant protein at a concentration of 0.5 μg/mL overnight at 4°C. The plates were washed with PBS-T (PBS + 0.05% Tween 20) solution 5 times. Each well was sealed with 300 μL sealing solution (PBS+2% BSA+5% milk) and incubated at 37°C for 2 h. Human plasma was initially diluted at 1:100, followed by 8 five-fold dilutions with a monoclonal diluent. Horseradish peroxidase-labeled goat anti-human IgG antibody was diluted at 1:5000 with a second antibody diluent. Color development occurred at 37°C for 10 min, and the reaction was terminated with 50 μL/well of 1 nm sulfuric acid solution. Detection was performed at 450 nm/630 nm using a dual wavelength enzymoscope.

**Statistical analysis**

The IgG antibody titres for both SARS-CoV-2 and seasonal HCoVs were Log10-transformed to improve linearity. Descriptive analysis was conducted using frequencies and median (interquartile range) quartiles. Statistical comparisons were made using two-tailed, nonparametric Mann–Whitney U-tests and two-tailed, nonparametric Dunn’s or Uncorrected Dunn’s Kruskal–Wallis tests for numerical data. Associations between groups were examined using Spearman’s rank correlation test. A two-sided P-value < 0.05 was considered statistically significant. Data analysis and visualization were performed using GraphPad Prism (version 9.0.0) and SPSS (version 26.0).
